# The neutrophil-lymphocyte ratio predicts all-cause and cardiovascular mortality among U.S. adults with rheumatoid arthritis: results from NHANES 1999-2020

**DOI:** 10.3389/fimmu.2023.1309835

**Published:** 2023-11-17

**Authors:** Erye Zhou, Jian Wu, Xin Zhou, Yufeng Yin

**Affiliations:** Department of Rheumatology and Immunology, The First Affiliated Hospital of Soochow University, Suzhou, Jiangsu, China

**Keywords:** neutrophil, lymphocyte, inflammation, mortality, rheumatoid arthritis

## Abstract

**Background:**

The neutrophil-to-lymphocyte ratio (NLR) is recognized as a biomarker for systemic inflammation and immune activation. However, its connection with the mortality risk in individuals with rheumatoid arthritis (RA) is not well understood. This study aimed to investigate the association between NLR and all-cause and cardiovascular mortality risk in U.S. adults with RA.

**Methods:**

Data were gathered from the National Health and Nutrition Examination Survey (NHANES) cycles spanning 1999 to March 2020. We included adults aged ≥20 years. The NLR was computed by dividing the neutrophil count by the lymphocyte count from complete blood counts. The maximally selected rank statistics method helped identify the optimal NLR cutoff value associated with significant survival outcomes. Multivariable logistic regression models were performed to investigate the relationship between the NLR and the all-cause and cardiovascular mortality of RA. Restricted cubic spline (RCS) analyses were utilized to detect whether there were linear or non-linear relationships between NLR and mortality.

**Results:**

In this study, 2002 adults with RA were included, with 339 having a higher NLR (≥3.28) and 1663 having a lower NLR (<3.28). During a median follow-up of 84 months, 79 RA individuals died. Participants with higher NLR had a 2-fold increased risk of all-cause (HR = 2.02, 95% CI: 1.53-2.66) and cardiovascular mortality (HR = 2.48, 95% CI: 1.34-4.57) versus lower NLR, after adjusting for demographics, socioeconomic status, and lifestyle factors. Kaplan-Meier analysis revealed that the survival rate for the higher NLR group was significantly lower than the lower NLR group, in terms of both all-cause and cardiovascular mortality (both P<0.0001). The RCS curve demonstrated a positive linear association between the NLR and all-cause and cardiovascular mortality.

**Conclusion:**

A higher NLR was independently predictive of elevated long-term mortality risk in U.S. adults with RA. The NLR may serve as an inexpensive, widely available prognostic marker in RA.

## 1 Introduction

Rheumatoid arthritis (RA) is a chronic autoimmune disease characterized by systemic inflammation that can lead to joint damage and disability (1). RA is a global public health concern, with prevalence estimates of 17.6 million cases worldwide in 2020, reflecting a 14.1% increase since 1990. Projections indicate that the RA burden will continue to rise, with forecasts predicting that the number of individuals affected globally will reach 31.7 million by 2050 (2). Patients with RA have a reduced life expectancy, with cardiovascular disease being the leading cause of premature mortality (3, 4). Understanding the factors influencing RA incidence and mortality is crucial for effective management and intervention strategies. Persistent inflammation in RA plays a crucial role in its pathogenesis and progression and is also linked to the increased cardiovascular risk and mortality observed in RA (4, 5). Therefore, markers of inflammation may have prognostic utility in this population.

A perfect prognostic scoring system should offer prognostic parameters that are easily discernible during diagnosis and be cost-effective in clinical practice, ensuring accessibility and affordability for widespread use. The neutrophil-to-lymphocyte ratio (NLR) stands as a burgeoning biomarker, offering insights into systemic inflammation and immune system activation (6). It provides a comprehensive perspective on both innate (neutrophils) and adaptive (lymphocytes) immune responses, showcasing its potential as an essential indicator in understanding the body’s inflammatory and immune states (7). Additionally, the NLR can be easily calculated from routine complete blood counts, making it highly accessible for clinical use. Its association with unfavorable outcomes has been demonstrated across a spectrum of diseases (8, 9).

In RA, previous studies have touched upon the relationship between NLR and the presence of the disease (10, 11). Recently, there has been a surge in studies exploring the potential of these hematologically derived biomarkers to differentiate between RA patients with active disease and those in remission (12–14). However, these investigations were often limited by small sample sizes. Additionally, while these studies explored the connection between NLR and RA incidence or disease activity, there is a notable gap in the literature regarding the association of NLR with the survival status of RA patients.

In light of the necessity for affordable and accessible prognostic markers in RA, our objective was to investigate the association between NLR and the risk of all-cause and cardiovascular mortality. We conducted these analyses using a vast, nationally representative sample of U.S. adults with RA obtained from the National Health and Nutrition Examination Survey (NHANES) dataset.

## 2 Methods

### 2.1 Study design and population

NHANES is an ongoing program conducted by the Centers for Disease Control and Prevention (CDC) of America to assess the health and nutritional status of US adults and children. The survey combines interviews, physical examinations, and laboratory tests. Detailed methods can be extracted from the official website (http://www.cdc.gov/nchs/nhanes.htm, accessed on 1 October 2023).

The original survey protocol underwent rigorous ethical scrutiny and received approval from the Institutional Review Board of the CDC. Prior to their participation, all individuals involved in the study willingly provided informed consent by signing consent forms. The present study, which utilized data derived from the approved survey, was reviewed by the Institutional Review Board of our center. Following careful evaluation, the Board determined that the present study fell under the category of exemption, signifying that it met the necessary ethical standards and regulations and therefore did not require additional ethical approval.

**Figure 1:**
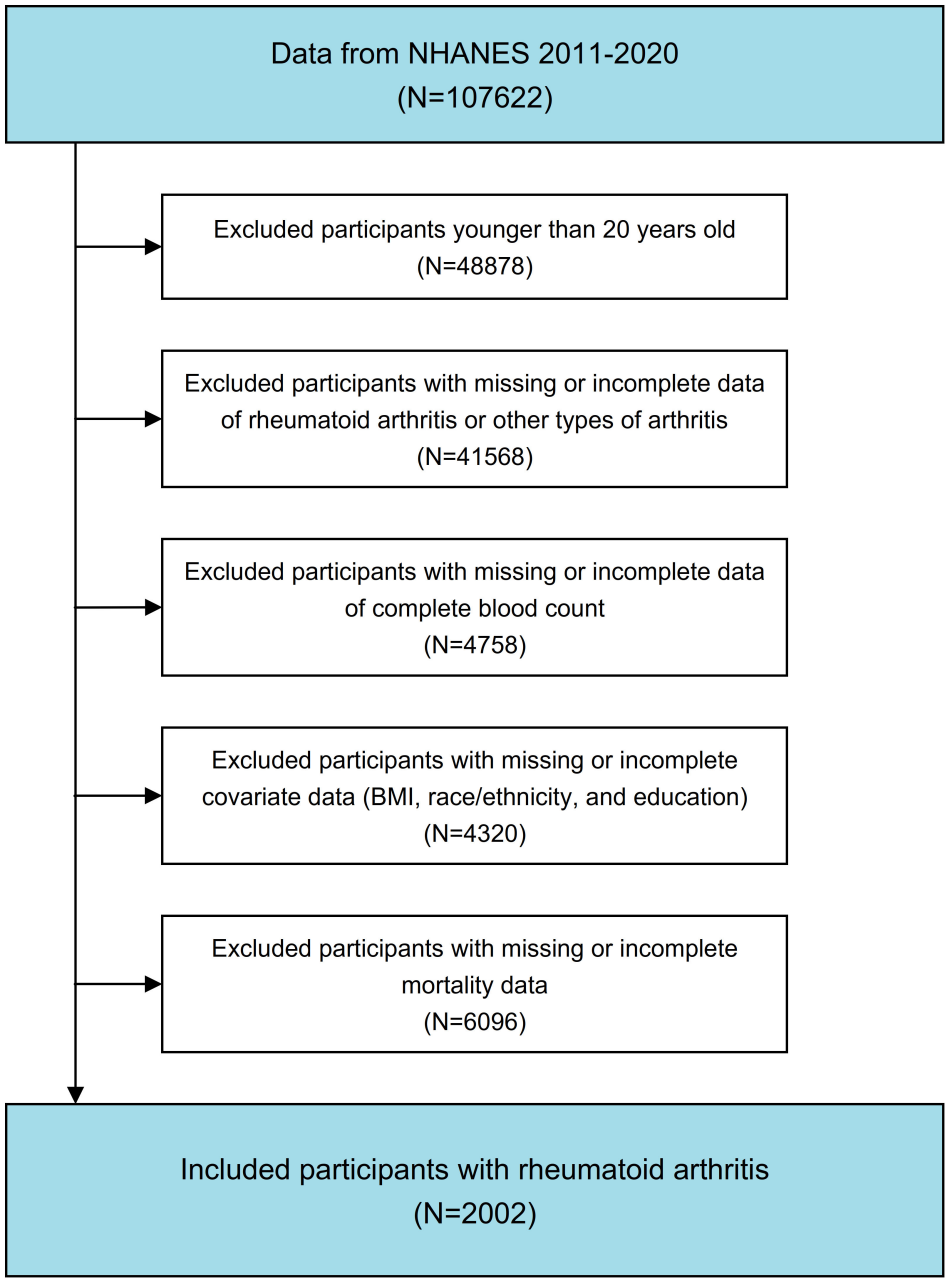
Flow chart portraying participant inclusion and exclusion in the current study.

This prospective cohort study utilized data from the continuous NHANES conducted between 1999 and March 2020. Exclusion criteria encompassed individuals younger than 20 years, those with incomplete data regarding complete blood count parameters, participants lacking information on RA or other forms of arthritis, and individuals with missing data for other essential covariates. The flowchart of participates inclusion and exclusion is depicted in **Figure 1**.

### 2.2 Definition of primary variates

Participants were specifically asked “Has a doctor or other health professional ever told you that you had arthritis?” and they could respond with either “Yes” or “No.” To further differentiate between types of arthritis, individuals with a positive response were asked to specify the type of arthritis, with response options including “Rheumatoid arthritis,” “Osteoarthritis,” “Psoriatic arthritis,” “Other,” “Refused,” and “Do not know.”

**Figure 2:**
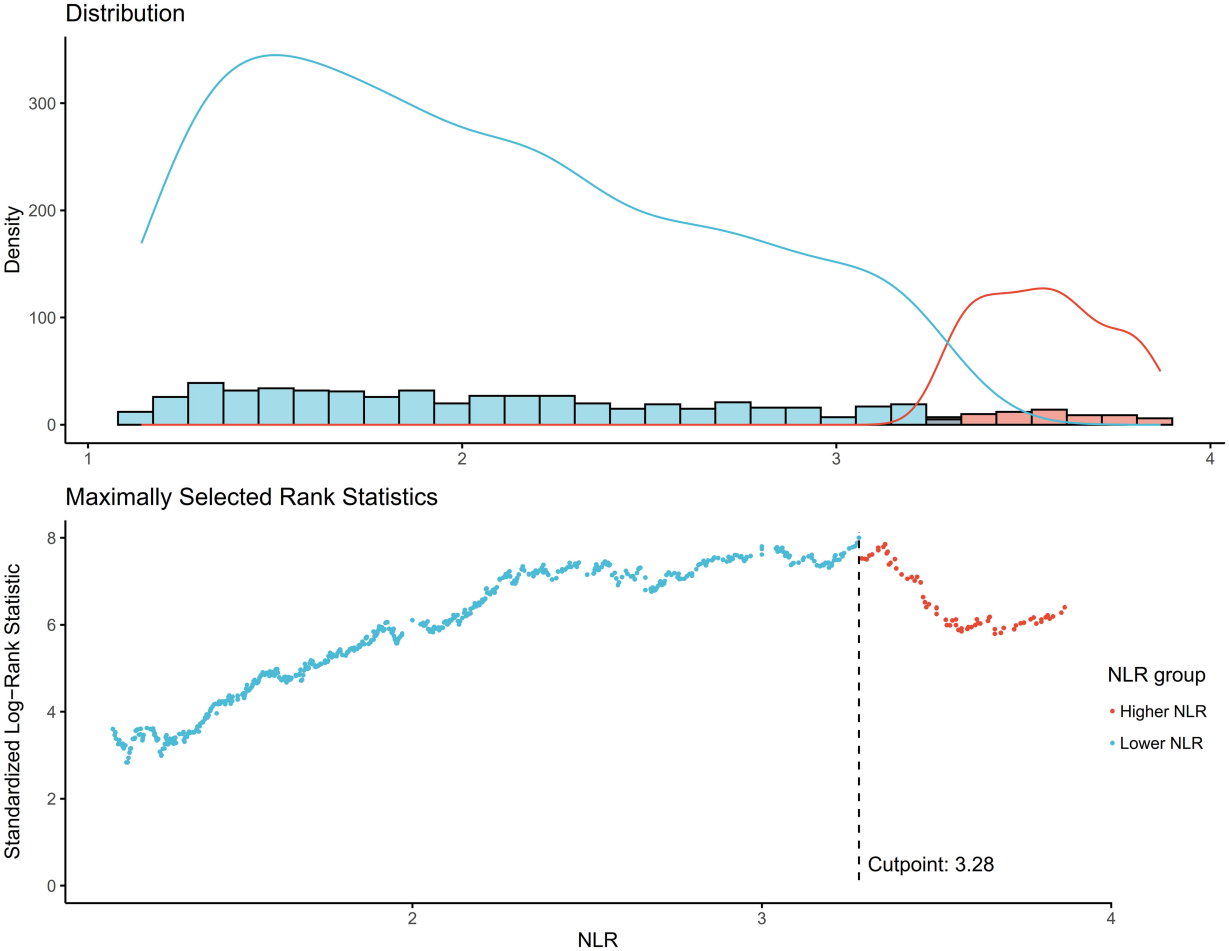
Determination of the NLR cutoff point using maximally selected rank statistics. Standardized Log-Rank Statistic was utilized in the calculation.

The NLR was calculated by dividing the absolute neutrophil count by the absolute lymphocyte count from the same automated complete blood count sample. The optimal NLR cutoff point of 3.28 for mortality prediction was determined using the maximally selected rank statistics method (**Figure 2**).

The primary outcome was all-cause mortality, ascertained using mortality-linked files (MLFs) documenting death from any cause. These mortality data were sourced from the National Death Index (NDI) database, accessible at https://www.cdc.gov/nchs/data-linkage/mortality-public.htm. Each participant’s follow-up duration was calculated from their date of participation until the date of death or until December 31, 2019, which marked the final update date of the NDI database.

### 2.3 Assessment of covariates

Demographic covariates included age (continuous), sex (male/female), and race/ethnicity (non-Hispanic white, non-Hispanic black, Mexican American, other Hispanic, and other/mixed race). Socioeconomic status was assessed using educational attainment (less than 9th grade, 9-11th grade, high school graduate, some college, college graduate and above) and poverty-income ratio (<1.29, 1.30-3.49, ≥3.50). Lifestyle and health-related factors comprised body mass index (BMI) categorized as underweight (<18.49 kg/m^2^), normal (18.50-24.99 kg/m^2^), overweight (25.00-29.99 kg/m^2^), or obese (≥30.00 kg/m^2^); smoking status (current smoker vs nonsmoker); and health insurance status (insured vs uninsured). All multivariable models sequentially adjusted for demographic factors, socioeconomics, lifestyle factors, comorbidities, and medications where appropriate. Missing covariate data were addressed through listwise deletion.

### 2.4 Statistical analysis

Baseline characteristics were summarized using the mean (standard deviation) for continuous variables and the number (percentage) for categorical variables. Differences by the presence of RA or NLR groups were compared using chi-squared tests for categorical variables and t tests for continuous variables. Cox proportional hazards models were used to investigate the association between NLR and all-cause and cardiovascular mortality in individuals with RA. Three models were constructed with incremental adjustment for potential confounders. Model 1 was unadjusted. In model 2, age, sex, race/ethnicity, and educational attainment were additionally adjusted; Model 3 was additionally adjusted by age, sex, race/ethnicity, educational attainment, BMI, poverty income ratio, smoking status, and health insurance. Kaplan-Meier curves were created to illustrate the survival probabilities based on different NLR categories. Subgroup analyses were performed to assess effect modification by pertinent clinical confounders. Restricted cubic spline (RCS) model was constructed to visualize the relationship between continuous NLR and outcomes. Analyses were performed using R version 4.3.1 (R Foundation for Statistical Computing, Vienna, Austria). Two-sided P<0.05 was considered statistically significant.

## 3 Results

### 3.1 Study population

Of 2002 participants with RA, 339 had higher NLR (≥3.28) while 1663 had lower NLR (<3.28) (**Table 1**). Participants in the higher NLR group were older than those in the lower NLR group (mean age 62.19 vs 55.48 years, P<0.001). The proportion of individuals aged 60-79 years was also higher in the higher NLR group compared to the lower NLR group (47.13% % vs 33.73%, P<0.001). The higher NLR group had a significantly greater proportion of non-Hispanic Whites compared to the lower NLR group (75.66% vs 67.57%, P<0.001). There were no significant differences between the higher and lower NLR groups in terms of educational attainment, poverty income ratio, BMI, waist circumference, smoking status, and health insurance (all P>0.05). The mean NLR was significantly higher in the higher NLR group compared to the lower NLR group (4.66 vs 1.91, P<0.001). These results indicate that NLR levels are associated with age and race/ethnicity, but not with socioeconomic factors, lifestyle factors, or other health-related variables. Participants with higher NLR were older and more likely to be non-Hispanic White.

**Table 1:** Basic characteristics of participates with RA based on NLR category.

Characteristic	Overall (n=2002)	Higher NLR (n=339)	Lower NLR (n=1663)	P value
Weighted No.	44,689,857	7,531,516	37,158,342	
Age (year)	56.61 (14.43)	62.19 (14.72)	55.48 (14.11)	<0.001
Age group				<0.001
20-39 years	161 (11.56%)	20 (6.63%)	141 (12.56%)	
40-59 years	641 (41.06%)	73 (27.58%)	568 (43.79%)	
60-79 years	941 (35.99%)	174 (47.13%)	767 (33.73%)	
≥80 years	259 (11.39%)	72 (18.66%)	187 (9.91%)	
Sex				0.11
Female	1,173 (58.74%)	170 (53.05%)	1,003 (59.89%)	
Male	829 (41.26%)	169 (46.95%)	660 (40.11%)	
Race/ethnicity				<0.001
Mexican American	324 (5.89%)	49 (6.09%)	275 (5.85%)	
Other Hispanic	154 (4.85%)	25 (4.68%)	129 (4.88%)	
Non-Hispanic White	878 (68.93%)	192 (75.66%)	686 (67.57%)	
Non-Hispanic Black	569 (15.98%)	54 (7.48%)	515 (17.70%)	
Other/multiracial	77 (4.35%)	19 (6.09%)	58 (3.99%)	
Educational attainment				0.7
Less than 9th grade	349 (9.56%)	54 (9.98%)	295 (9.47%)	
9-11th grade	406 (17.41%)	61 (16.18%)	345 (17.66%)	
High school graduate	482 (28.10%)	91 (29.89%)	391 (27.74%)	
Some college	544 (31.21%)	92 (32.93%)	452 (30.86%)	
College graduate or above	221 (13.72%)	41 (11.02%)	180 (14.27%)	
Poverty income ratio	2.52 (1.58)	2.36 (1.51)	2.55 (1.60)	0.2
Poverty income ratio group				0.061
0-1.29	807 (31.03%)	128 (32.49%)	679 (30.73%)	
1.30-3.49	749 (37.62%)	140 (42.79%)	609 (36.57%)	
≥3.50	446 (31.35%)	71 (24.72%)	375 (32.70%)	
BMI (kg/m^2^)	30.31 (7.37)	29.79 (7.38)	30.41 (7.37)	0.2
BMI group				0.3
Underweight	23 (1.24%)	4 (2.35%)	19 (1.01%)	
Normal	423 (22.80%)	90 (26.19%)	333 (22.12%)	
Overweight	613 (29.71%)	99 (29.71%)	514 (29.71%)	
Obese	943 (46.25%)	146 (41.75%)	797 (47.16%)	
Waist circumference (cm)	103.03 (16.96)	103.52 (17.49)	102.93 (16.85)	>0.9
Smoking status				0.3
Non-smoker	868 (40.04%)	148 (36.76%)	720 (40.71%)	
Smoker	1,134 (59.96%)	191 (63.24%)	943 (59.29%)	
Health insurance				0.7
Insured	1,751 (87.99%)	302 (87.23%)	1,449 (88.14%)	
Uninsured	251 (12.01%)	37 (12.77%)	214 (11.86%)	
NLR	2.38 (1.41)	4.66 (1.89)	1.91 (0.63)	<0.001

Continuous variables are expressed as mean ± standard deviation (SD), while categorical variables are presented as number (%). All estimates were adjusted for sample weights and complex survey designs, with means and percentages further adjusted for the survey weights of NHANES. RA, rheumatoid arthritis; BMI, body mass index; NLR, neutrophil to lymphocyte ratio.

### 3.2 Association between NLR and mortality

**Figure 3:**
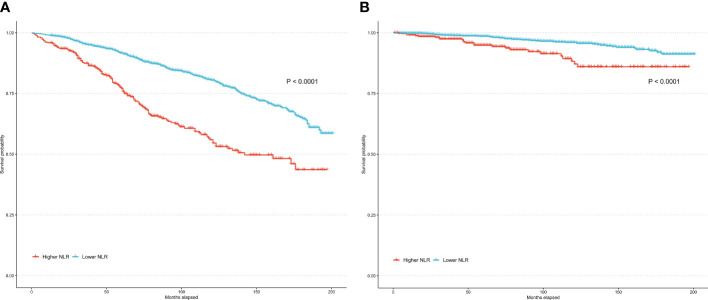
Kaplan–Meier curves of all-cause mortality **(A)** and cardiovascular mortality **(B)** among participates with RA.

Over a median follow-up of 84 months, 79 RA individuals died. The higher NLR group had 24 observed deaths versus only 11.7 expected deaths. In contrast, the lower NLR group had fewer observed deaths (55) than expected (67.3). In the unadjusted model (Model 1), higher NLR as a continuous variable was significantly associated with increased risk of all-cause mortality (HR per 1 unit increase 1.24, 95% CI: 1.17-1.31, P<0.001) and cardiovascular mortality (HR = 1.29, 95% CI: 1.18-1.40, P<0.001). When NLR was evaluated as a categorical variable, participants in the higher NLR group had a 2-fold higher risk of all-cause mortality (HR = 2.11, 95% CI: 1.59-2.81, P<0.001) and a 3-fold higher risk of cardiovascular mortality (HR = 3.23, 95% CI: 1.82-5.72, P<0.001) compared to the lower NLR group. Model 2 was adjusted for demographic factors including age, sex, race/ethnicity, and education. The associations remained statistically significant. Model 3 was further adjusted for BMI, poverty income ratio, smoking, and insurance in addition to demographics. The positive associations between higher NLR and elevated mortality risks persisted. As a continuous variable, each 1-unit NLR increase was linked to 20% and 22% higher all-cause and cardiovascular mortality. Categorically, the higher NLR group had 2.02 and 2.48 times the risk of all-cause and cardiovascular mortality versus lower NLR participants (**Table 2**). Kaplan-Meier analysis revealed that the survival rate for the higher NLR group was significantly lower than the lower NLR group, in terms of both all-cause and cardiovascular mortality (both P<0.0001) (**Figure 3**). These findings suggest that higher NLR, whether evaluated as a continuous or categorical variable, was consistently and independently associated with increased all-cause and cardiovascular mortality risk in US adults with RA.

**Table 2:** Association of NLR with mortality risk in adults with RA.

Characteristic	Model 1		Model 2		Model 3	
	HR (95% CI)	P value	HR (95% CI)	P value	HR (95% CI)	P value
All-cause mortality						
NLR (continuous variable)	1.24 (1.17, 1.31)	<0.001	1.21 (1.14, 1.29)	<0.001	1.20 (1.14, 1.26)	<0.001
Higher NLR*	2.11 (1.59, 2.81)	<0.001	2.11 (1.59, 2.81)	<0.001	2.02 (1.53, 2.66)	<0.001
Cardiovascular mortality						
NLR (continuous variable)	1.29 (1.18, 1.40)	<0.001	1.26 (1.15, 1.38)	<0.001	1.22 (1.13, 1.31)	<0.001
Higher NLR*	3.23 (1.82, 5.72)	<0.001	2.66 (1.43, 4.96)	0.002	2.48 (1.34, 4.57)	0.004

In model 1, covariates were not adjusted; Model 2 was adjusted by age, sex, race/ethnicity, and educational attainment; Model 3 was adjusted by age, sex, race/ethnicity, educational attainment, BMI, poverty income ratio, smoking status, and health insurance. *Risk of mortality was analyzed compared to lower NLR group.

### 3.3 Stratified analyses

In subgroup analyses stratified by sex, age, race/ethnicity, education, BMI, and smoking status, higher NLR was consistently associated with increased all-cause and cardiovascular mortality risk across all subgroups (**Table 3**). For all-cause mortality, no significant interaction was observed by sex (P=0.659), age (P=0.536), race/ethnicity (P=0.863), education (P=0.735), BMI (P=0.873), or smoking status (P=0.277). For cardiovascular mortality, a significant interaction was found by race/ethnicity (P=0.014). No other significant interactions were detected. These results indicate that the relationship between higher NLR and increased mortality risk persists across subgroups of potential confounders. The racial/ethnic difference in the association between NLR and cardiovascular mortality warrants further investigation.

**Table 3:** Subgroup analyses of NLR and mortality risk in RA.

Variable	All-cause mortality			Cardiovascular mortality		
	HR (95% CI)	P value	P for inter- action	HR (95% CI)	P value	P for inter- action
Overall	2.44 (1.97, 3.01)	<0.001		2.52 (1.56, 4.07)	<0.001	
Sex			0.659			0.684
Female	2.53 (1.87, 3.44)	<0.001		2.08 (0.94, 4.58)	0.07	
Male	2.29 (1.7, 3.09)	<0.001		2.62 (1.42, 4.85)	0.002	
Age group			0.536			0.927
<60 years	1.71 (0.83, 3.53)	0.144		1.85 (0.21, 16.55)	0.582	
≥60 years	2.15 (1.72, 2.69)	<0.001		2.13 (1.3, 3.49)	0.003	
Race/ethnicity			0.863			0.014
White	2.33 (1.8, 3.02)	<0.001		3.73 (2.14, 6.51)	<0.001	
Other	2.24 (1.53, 3.28)	<0.001		0.29 (0.04, 2.16)	0.229	
Educational attainment			0.735			0.683
Less than 9th grade	2.00 (1.24, 3.22)	0.004		1.63 (0.55, 4.86)	0.378	
9-11th grade	2.99 (1.86, 4.8)	<0.001		2.46 (0.8, 7.55)	0.116	
High school graduate	2.46 (1.67, 3.61)	<0.001		3.22 (1.44, 7.17)	0.004	
Some college	2.69 (1.63, 4.46)	<0.001		2.25 (0.58, 8.72)	0.239	
College graduate or above	2.58 (1.3, 5.12)	0.007		6.33 (1.05, 38.02)	0.044	
BMI group			0.873			0.753
Underweight	5.32 (0.74, 38.12)	0.096		4.87 (0.3, 77.93)	0.263	
Normal	2.29 (1.54, 3.39)	<0.001		3.21 (1.43, 7.25)	0.005	
Overweight	2.54 (1.77, 3.65)	<0.001		2.00 (0.65, 6.15)	0.226	
Obese	2.36 (1.64, 3.4)	<0.001		2.13 (1, 4.54)	0.05	
Smoking status			0.277			0.451
Smoker	2.20 (1.67, 2.9)	<0.001		2.15 (1.13, 4.09)	0.019	
Non-smoker	2.83 (2.02, 3.95)	<0.001		3.14 (1.52, 6.5)	0.002	

HRs were adjusted for age, sex, race/ethnicity, BMI, and smoking status. When analyzing age, adjustments were made for the other variables, and so on for subsequent analyses.

### 3.4 Nonlinear relationship of NPR and mortality

**Figure 4:**
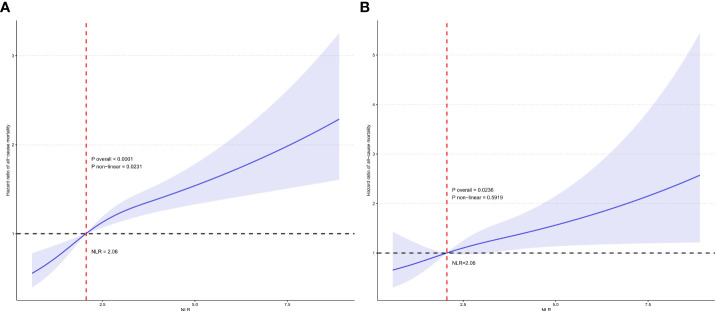
Restricted cubic spline (RCS) illustrating the relationship between NLR and all-cause mortality **(A)** and cardiovascular mortality **(B)** among participates with RA.

**Figure 4A** displays the RCS curve for the association between continuous NLR and all-cause mortality risk in participates with RA. The overall association was statistically significant (P overall <0.0001). There was evidence of a nonlinear relationship, with the curve showing an initial steep increase in mortality risk at lower NLR values which plateaued at higher values (P non-linear = 0.0231). **Figure 4B** shows the RCS curve for the relationship between continuous NLR and cardiovascular mortality risk in participates with RA. The overall association was statistically significant (P overall = 0.0236). The curve depicts a linear relationship between increasing NLR and cardiovascular mortality without statistical evidence of nonlinearity (P non-linear = 0.5919). These findings highlight the differential relationships between this inflammatory biomarker and various mortality outcomes in individuals with RA.

## 4 Discussion

In this large nationally representative cohort of US adults, we found that a higher NLR was independently predictive of increased all-cause and cardiovascular mortality risk among adults with RA. Kaplan-Meier curves illustrated significantly lower survival probabilities for those with higher NLR. RCS analyses demonstrated linear positive associations between NLR and all-cause and cardiovascular mortality. These data highlight the potential utility of the NLR as an inexpensive and widely available marker for RA risk stratification and prognostication in clinical practice.

A number of prior studies have examined the association between NLR and RA with inconsistent findings. Several studies found significantly higher NLR values in RA patients than in healthy controls, suggesting that the NLR may be a useful biomarker for RA diagnosis (15–18). Other studies did not observe significant differences in NLR between RA and controls, implying limited diagnostic utility (18, 19). Regarding the relationship between the NLR and RA disease activity, most studies reported positive correlations, with a higher NLR associated with more active disease and an even lower response rate after TNF-α therapy or an increased risk of anti-TNF-α agent withdrawal due to a lack of efficacy (12, 14, 20–24). This indicates that the NLR could serve as an inflammatory marker reflecting RA disease activity. Compared to prior studies with small sample sizes, our study leveraged a large nationally representative cohort to provide robust evidence for the utility of the NLR as a biomarker for RA risk stratification and prognostication.

Prior studies have reported associations between higher NLR and increased mortality risk across the general population and various conditions marked by significant inflammatory components (25, 26). In glomerulonephritis, a study established a connection between NLR and mortality prediction in patients with rapidly progressive glomerulonephritis (RPGN). It was found to be associated with systemic inflammation and exhibited a negative correlation with the percentage of fibro-cellular crescents (27). In asthma, a previous study concluded that the NLR and other complete blood cell count-derived inflammatory biomarkers are associated with a higher risk of all-cause and respiratory disease mortality in adult patients (28). In colon cancer, a study found that the NLR was an independent predictor of overall mortality, with the authors concluding that the NLR may serve as a useful prognostic marker (29). Similarly, in acute coronary syndrome (ACS), a meta-analysis of over 9,000 patients demonstrated that an elevated pretreatment NLR predicted higher medium- to long-term mortality and major adverse cardiac events (MACEs) (30, 31). More recently, in COVID-19, multiple studies have shown that the NLR correlates with disease severity and mortality (32, 33). A meta-analysis concluded that evaluating the NLR could help clinicians identify severe COVID-19 cases early and initiate timely management to potentially reduce mortality (26). However, the available evidence regarding the prognostic significance of NLR for survival in RA remains limited. Our study effectively addresses this gap, providing new evidence that underscores the significance of the NLR as an independent predictor of all-cause mortality in RA.

Moreover, determining specific NLR cutoffs tailored to individual patient populations could improve the predictive accuracy of this marker in clinical settings. While a consensus on normal NLR values is lacking, there is a suggested range of 1–2 for normal values, 2–3 as a gray area indicating subclinical inflammation, and values above 3 indicating inflammation (34). Our study aligns with these recommendations, establishing an optimal NLR cutoff of 3.28, which correlated with the most significant survival outcomes. However, as noted in other conditions, the NLR alone without other inflammatory markers may have limited utility. Future research should examine combining NLR with other biomarkers to improve prognostic accuracy in RA.

The intricate pathological processes underlying autoimmune diseases have been meticulously explored, elucidating the interplay between innate and adaptive immunity. The NLR, comprising neutrophil and lymphocyte counts, provides valuable insights into the body’s inflammatory status and immune response. A higher neutrophil count suggests an ongoing nonspecific inflammatory process, while a lower lymphocyte count indicates a relatively compromised immune system (35). Several lines of evidence suggest that dysregulated innate immunity and inflammation play key roles in RA pathogenesis (36, 37). Neutrophils are among the first responders during inflammation and immune activation in RA (37). Additionally, neutrophils may function as antigen-presenting cells (APCs) in presenting antigens and activating T cells to perpetuate chronic inflammation and autoimmunity (38). They also produce proteases such as elastase that contribute to cartilage and bone destruction in RA (39). Therefore, an elevated NLR likely implies higher activation of neutrophils and innate inflammatory pathways contributing to RA development and progression. The increased systemic inflammation marked by a high NLR could also accelerate atherosclerosis, conferring greater cardiovascular risk and mortality (40). Conversely, lymphocytes, such as regulatory T cells, normally act to suppress inflammatory responses and restore immune homeostasis (41). A lower lymphocyte count signified by a higher NLR may indicate impaired anti-inflammatory immunity. In summary, an elevated NLR represents enhanced proinflammatory innate responses along with potential deficiencies in anti-inflammatory lymphocyte activity. This imbalance could promote the inflammatory and immunopathogenic mechanisms underlying RA onset and severity. The NLR provides a simple readout of the skewing between pathological innate immunity and protective adaptive responses in RA.

Several limitations should be noted. First, the observational design of this study limit causal inferential ability. We identified associations between NLR and outcomes but cannot confirm a definitive causal relationship. Second, residual confounding is possible despite adjustments for an extensive set of potential confounders. Unmeasured factors such as comorbidities, smoking intensity, medications, and RA characteristics could influence the observed relationships. Third, the NLR was measured at a single timepoint and may not reflect variability over time or with interventions. Serial NLR measurements could provide a more accurate representation of inflammatory status. Finally, the study population comprised U.S. adults, which may restrict generalizability to other populations. Further studies in diverse cohorts are warranted to validate our findings. However, the strengths of a large nationally representative sample, long follow-up, and robust adjustment for confounders enhance the reliability of the results.

## 5 Conclusion

In summary, the NLR provides a simple readout of the balance between pathological innate inflammation and protective adaptive immunity. Our study of a large nationally representative cohort demonstrates that a higher NLR is independently predictive of increased risks of both all-cause and cardiovascular mortality among adults with RA. These findings highlight the potential clinical utility of the NLR as an inexpensive and widely available biomarker that can be integrated into routine care to improve risk stratification and prognostication in RA patients.

## Data availability statement

The original contributions presented in the study are included in the article/supplementary materials, further inquiries can be directed to the corresponding author/s.

## Ethics statement

The original survey protocol underwent rigorous ethical scrutiny and received approval from the Institutional Review Board of CDC. The studies were conducted in accordance with the local legislation and institutional requirements. Written informed consent for participation in this study was provided by the participants’ legal guardians/next of kin.

## Author contributions

JW: Conceptualization, Writing – review & editing. EZ: Data curation, Writing – original draft. XZ: Methodology, Writing – review & editing. YY: Supervision, Writing – original draft, Writing – review & editing.
